# Condition-dependent virulence of slow bee paralysis virus in *Bombus terrestris*: are the impacts of honeybee viruses in wild pollinators underestimated?

**DOI:** 10.1007/s00442-017-3851-2

**Published:** 2017-03-30

**Authors:** Robyn Manley, Mike Boots, Lena Wilfert

**Affiliations:** 1 Centre for Ecology and Conservation, University of Exeter, Penryn Campus, Penryn, TR10 9EF UK; 2 Department of Integrative Biology, University of California, Berkeley, 1005 Valley Life Sciences Building #3140, Berkeley, CA 94720-3140 USA

**Keywords:** Pollinators, Virulence, Virus, Bees, Longevity

## Abstract

Slow bee paralysis virus (SBPV)—previously considered an obligate honeybee disease—is now known to be prevalent in bumblebee species. SBPV is highly virulent in honeybees in association with *Varroa* mites, but has been considered relatively benign otherwise. However, condition-dependent pathogens can appear asymptomatic under good, resource abundant conditions, and negative impacts on host fitness may only become apparent when under stressful or resource-limited conditions. We tested whether SBPV expresses condition-dependent virulence in its bumblebee host, *Bombus terrestris,* by orally inoculating bees with SBPV and recording longevity under satiated and starvation conditions. SBPV infection resulted in significant virulence under starvation conditions, with infected bees 1.6 times more likely to die at any given time point (a median of 2.3 h earlier than uninfected bees), whereas there was no effect under satiated conditions. This demonstrates clear condition-dependent virulence for SBPV in *B. terrestris.* Infections that appear asymptomatic in non-stressful laboratory assays may nevertheless have significant impacts under natural conditions in the wild. For multi-host pathogens such as SBPV, the use of sentinel host species in laboratory assays may further lead to the underestimation of pathogen impacts on other species in nature. In this case the impact of ‘honeybee viruses’ on wild pollinators may be underestimated, with detrimental effects on conservation and food security. Our results highlight the importance of multiple assays and multiple host species when testing for virulence, in order for laboratory studies to accurately inform conservation policy and mitigate disease impacts in wild pollinators.

**Electronic supplementary material:**

The online version of this article (doi:10.1007/s00442-017-3851-2) contains supplementary material, which is available to authorized users.

## Introduction

Emerging infectious diseases are one of several key factors linked to pollinator decline (Manley et al. 2015; Vanbergen et al. 2013). Several fast-evolving Picorna-like RNA viruses, known to be pathogenic to honeybees (Carreck et al. 2010; Cox-Foster 2007; Genersch et al. 2010) have a broad host range, threatening ecologically and economically important wild pollinators (Manley et al. 2015; McMahon et al. 2015). Recent large-scale field studies have found deformed wing virus (DWV), black queen cell virus (BQCV), acute bee paralysis (ABPV) and slow bee paralysis virus (SBPV) to be prevalent in bumblebee species (Fürst et al. 2014; McMahon et al. 2015). High disease prevalence in wild, unmanaged pollinators—who play an important role as an ecosystem service (e.g. Garibaldi et al. 2013)—has raised concerns over the impact of diseases and the risk of spillover from managed honeybees (Fürst et al. 2014; Manley et al. 2015; Vanbergen et al. 2013). However, with the exception of DWV (Fürst et al. 2014), Israeli acute paralysis virus (IAPV) and Kashmir bee virus (KBV) (Meeus et al. 2014), the actual impact of viral diseases on wild pollinators is unknown.

Here, we used a controlled infection study to examine the impact of SBPV in a natural host, the bumblebee *Bombus terrestris*. SBPV is a picornavirus (de Miranda et al. 2010) which was first discovered in 1974 in an English honeybee (*Apis mellifera*) population. However, this virus is common in a range of bumblebee species and at its highest prevalence in *B. hortorum* in the UK (McMahon et al. 2015). In *A. mellifera*, SBPV only causes high mortality in association with the recently emerged ectoparasitic mite *Varroa destructor,* while otherwise appearing asymptomatic (Carreck et al. 2010; Santillán-Galicia et al. 2014). SBPV causes paralysis in the two anterior pairs of legs 10 days after injection into the haemolymph (Bailey and Woods 1974). *Varroa*, which exclusively infests honeybees, feeds on hemolymph and can thus serve as a vector directly transmitting viral infections into the hemolymph. Field studies that have found virus-positive pollinator species have collected foraging individuals (Evison et al. 2012; Levitt et al. 2013; Singh et al. 2010). Recent UK-wide data finds on average 5% of apparently healthy foraging bumblebees harbouring SBPV, with more than a third of SBPV-positive individuals carrying high viral titres (>10^9^ virus particles per individual) (McMahon et al. 2015). Based on these field-surveys, it is clear that SBPV is found in nature but seems to exist asymptomatically.

Virulence in its broad sense is a measure of pathogen impact on host fitness. While some pathogens are highly virulent, imposing fitness costs on hosts that lead to mortality and reduced fecundity, others often seem to have no obvious impact. However, in seemingly benign pathogens, costs may be hidden if the host is able to compensate for the impacts through increased resource use, but revealed under starvation conditions when resources are too low to maintain defence costs (Moret and Schmid-Hempel 2000). Increased virulence under environmental stress is a common phenomenon across a range of pathogens and their hosts (Boots and Begon 1994; Jaenike et al. 1995; Jokela et al. 2005; Koella and Offenberg 1999; Restif and Kaltz 2006; Steinhaus 1958) and has been documented in honeybees and bumblebees (Brown et al. 2000; Goulson et al. 2015; Moret and Schmid-Hempel 2000). It is therefore important to examine the impact of pathogens across a range of host resource conditions.

Condition-dependent virulence has the potential to impact on individual bees, colonies and populations of wild pollinators, potentially contributing to population declines under poor environmental conditions. The availability of multiple reservoir species may also affect a pathogen’s virulence and mask the fitness effects of pathogen infection in wild hosts (Leggett et al. 2013). Moreover, in a multi-host-pathogen system there is potential to underestimate the impact of pathogens, as sentinel species used in lab studies may not be representative if they are assayed under conditions of resource superabundance. For example, *A. mellifera*, the sentinel species in many pesticide studies, are less susceptible to dietary imidacloprid, a systemic neonicotinoid, than *B. terrestris*; therefore raising concern about the true impact of this pesticide on wild pollinator populations (Cresswell et al. 2012). In a survey of honeybees and five bumblebee species in the UK, the mean prevalence of SBPV in *B. terrestris* was comparable to honeybees and other bumblebee species at ~6%, while *B. hortorum* had a significantly higher prevalence (McMahon et al. 2015), thus differences in host susceptibility and potentially host tolerances may exist. An understanding of the condition dependence of virulence, the impact on non-*apis* hosts, and the likely environmental changes that populations are experiencing is therefore critical to an assessment of the impact of these pathogens on wild pollinator communities.

## Materials and methods

We used three *B. terrestris* colonies (Biobest Belgium N.V.) in the experiments and kept them at 28 °C with ad libitum irradiated pollen (Biobest, gamma radiation) and sugar water. We used Invertbee feed sugar (*BelgoSuc*), which has a sugar content of 71.4%, and was diluted 1:1 with water. We confirmed the absence of four common bee viruses (deformed wing virus (DWV), black queen cell virus (BQCV), SBPV and acute bee paralysis virus (ABPV) (McMahon et al. 2015) by RT-PCR (see methods below and supplementary tables S1 and S2 for details) in ten bees from each colony (>20% of the young colony). Graystock et al. (2013) found that in colonies where DWV was present, >10% of bees within the colony were infected with the virus. Thus, screening >20% of the colony is sufficient to determine virus-free status. Phase-contrast microscopy of faeces and gut tissue samples from the same ten bees per colony confirmed the absence of the gut parasites *Nosema* spp, *Crithidia* spp and *Apicystis bombi*. Bees were handled throughout the experiment using forceps that were flamed between individuals.

To imitate a naturally occurring infection, we prepared virus inoculum from five naturally SBPV-infected wild-caught bees from Scotland (2× *B. hortorum* and 3× *B*. *pascuorum*), known to be uninfected by DWV, BQCV and ABPV by previous PCR. We homogenised their abdomen in 400 μl of insect ringer solution per bee and combined them into one inoculum solution. We prepared the control inoculum in the same way from uninfected colony bees. SBPV and control inocula were confirmed by RT-PCR to be SBPV positive and negative, respectively.

### Experimental set-up

#### Infection time course

To determine the pattern of SBPV infection in the bee guts over time, we collected 50 bees from each of the three colonies of random age and size. We confined all bees individually in tubes and starved them for 2 h before dosing them with 10 µl of sugar water solution, containing 5 µl of SBPV extract. The 10 µl droplet was pipetted onto the upper side of the tube and all bees were observed to drink the droplet until no liquid was visible. We then maintained bees individually with ad libitum pollen and sugar water. We killed three bees per colony every 2 days for 14 days, then once a week until day 28. Guts were dissected from each bee for qPCR analysis (see below). Full guts were used in this assay to track viral loads in the gut specifically, as faecal-oral transmission is thought to be a primary transmission route for insect viruses. Dose was determined from analysis of day zero bees (killed 2 h post dosing) to be directly comparable to the other time points.

#### Longevity under satiated conditions

We collected 142 newly emerged worker bees over 4 days from the same three colonies (46, 59 and 37, respectively, from colonies A, B and C). It was essential to use same-age bees when comparing time to death between treatment groups under satiated conditions because of the bees’s long lifespan (up to 3 months). Gut fauna was reconstituted by feeding all bees a 10 µl preparation of faeces mixed with sugar water, which we collected from worker bees in the corresponding colony (Koch and Schmid-Hempel 2011). At 6 days old, we dosed and maintained the bees as detailed above. The experiment continued for 95 days, by which point 94% of bees had died; we killed the eight surviving bees. We collected dead bees daily and stored them at −80 °C .

#### Longevity under starvation conditions

It is paramount to use bees from the same colonies in each assay, as colony-specific gut microbiota play a role in immunity (Koch and Schmid-Hempel 2012). Thus, to reach a sufficient sample size we collected 150 worker bees (age range 0–3 week-old) from the same three colonies (50 per colony); bees were randomly allocated to either the treatment or the control group, each individually fed with 10 µl of sugar water solution, containing either 5 µl of SBPV extract or control extract, respectively. To control for age and allow for the acquisition of the normal gut flora, all workers in the colonies were marked on a set day and experimental bees were then randomly chosen and allocated from unmarked bees that had emerged over a 3-week period to randomise age differences across treatments. We maintained workers individually with ad libitum pollen and sugar water. On day 10 post-infection, we starved all surviving individuals (*N* = 144) of pollen and sugar water and recorded time to death every 15 min until 56 h, at which point we killed the six surviving individuals. Death was confirmed by lack of movement when we turned the tubes at each 15 min time point.

### RNA extractions and RT-PCR

To allow for multiple assays, we cut all bees from the two longevity assays in half laterally. Guts were extracted from infection time course bees. One half bee, or gut extract, was used to determine infection status through RT-PCR. We extracted RNA individually from each sample using Trizol© (Invitrogen, Carlsbad, CA, USA) following the manufacturer’s instructions. Briefly, samples were homogenised with glass beads in 1.3 ml Trizol© in a tissue-lyser (note; 500 µl of Trizol was used for the guts). RNA was separated using bromo-chloropropane and precipitated in isopropanol. The RNA was washed with 75% ethanol and re-suspended in 400 µl of diethylpyrocarbonate (DEPC)-treated water. We converted 2 µl of RNA into first-strand cDNA using GoScript™ Reverse Transcriptase, according to the manufacturer’s instructions (Promega) using random hexamer primers and RNasin^®^ to avoid RNA degradation. SBPV positive RNA and water negatives were run as controls.

SBPV specific forward (5′-GAGATGGATMGRCCTGAAGG-3′) and reverse primers (5′-CATGAGCCCAKGARTGTGAA-3′) were used to amplify a 915 bp cDNA fragment by PCR. We designed primers based on the published Rothamsted strain coding region [EU035616 (de Miranda et al. 2010)]. We carried out PCR in 20 µl reactions using GoTaq^®^ DNA Polymerase, with 35 amplification cycles, an annealing temperature of 55 °C for 30 and 20 s of extension at 72 °C (table S1). Every run included a known positive SBPV sample and a water negative as controls. 5 µl of PCR product were run on 1.5% TAE agarose gel and RedSafe™ nucleic acid staining solution. In addition, we carried out PCR with arginine kinase (AK), a stable reference gene for *B. terrestris* (Horňáková et al. 2010), to check the quality of RNA extractions (primers: 5′-TGACAAGCATCCACCAAAAG-3′, 5′-TCGTCGATCAGTTTCTGCTG-3′), amplifying a 263 bp fragment using the PCR programme as above.

### Real-time quantitative RT-PCR

RNA extractions from all bee guts in the infection time course were measured on a Nanodrop 1000 spectrometer. 260/280 ratios ranged from 1.81 to 2.03 with 64% of samples falling between 1.9 and 2.03. Two samples fell below 1.8 and were excluded from qPCR analysis. All remaining samples were run on a Qubit^®^ 3.0 Fluorometer using the Qubit^®^ RNA quantification assay: RNA concentrations ranged from 1.8 to 207 ng/µl. This range was accounted for by diluting all sample RNA to 5 ng/µl (using RNase/DNase free water). Samples that were already at 5 ng/µl or below (*N* = 10) were used directly.

One-step absolute quantification of SBPV was carried out on the Step One ABI Applied Biosystems qPCR machine. Taqman^®^ primer and probe assays were designed and optimised by Primerdesign© for SBPV and an endogenous control bee gene, Actin beta (ACTB) (SBPV; Accession Number EU035616, context sequence length -138 and the anchor nucleotide—4189: and ACTB; Accession Number FN391379, context sequence length—211 bases and the anchor nucleotide—641).

Each sample was run in duplicate for both SBPV and ACTB assays on each plate, along with a no-template control, and a five point standard curve (1:10 dilution series) also run in duplicate. Reactions were carried out with Precision one-step mastermix and 2.5 μl of RNA in 12.5 µl reactions, according to the manufacturer’s instructions. Reverse transcription occurred at 42 °C for 10 min, enzyme activation took place at 95 °C for 8 min, followed by 40 cycles of amplification at 95 °C for 10 s and data collection at 60 °C for 1 min. The standard curve was created using a SBPV positive control provided by Primerdesign with a known quantity of 2 × 10^5^ viral particles per µl, thus SBPV could be quantified between 5 × 10^1^ and 5 × 10^5^ viral copies (figure S1). SBPV assay efficiency ranged from 90.3 to 92.4% across plates. Quantitation cycle (Cq) values and absolute viral quantities were calculated using StepOne software. ACTB Cq values were in the range of 17.4–24.8 Cq and stayed stable relative to SBPV Cq values (figure S2), confirming extraction of a valid biological template.

### Body size and fat content

For both longevity assays, we measured the length of the radial wing cell, using a Leica camera microscope and ImageJ, as a proxy for body size (Brown et al. 2000). For the starvation assay, we used the second half of each individual to measure fat content—as a proxy for condition—by adapting methods from Ellers (1996): the half abdomen were dried at 70 °C for 3 days and then weighed with a precision balance, before being placed in 2 ml of dichloromethane:methanol (2:1 mix) for 2 days, dried at 70 °C for another 3 days and weighed again. The difference between the two weights is taken as a proxy for the amount of fat. The relative fat content is the ratio of amount of fat (mg) and radial wing cell length (mm).

### Statistical analysis

We carried out all analyses in R v3.2.3. We used the *lme4* package (Bates et al. 2015) to run a GLMM (generalised linear mixed model) modelling log viral load as dependent on time post-infection and host colony, with qPCR plate (*N* = 7) as a random effect. *Lmertest* was used to determine significance. Model simplification, using term removal and Anova for model comparison, was used to determine the minimum adequate model of best fit. After model simplification, goodness of fit was determined using residual plots. One-sided Kolmogorov–Smirnov tests were used to determine significant differences in viral load between time points.

For survival analysis we used the *Survival* package (Therneau 2008). Possible confounding correlations between body size and relative fat content were tested and found to be insignificant. Kaplan–Meier survival curves were produced using the *survfit* function; the *survdiff* function was used to test the difference between curves with a log-rank test. We used the *coxph* function to determine the effects of infection status, body size and relative fat content on the survival of bees, with the frailty function used to fit colony as a random term in the survival models. We checked for correlations between fixed factors using the *cor.test* function. Model simplification, using term removal and Anova for model comparison, was used to determine the minimum adequate model of best fit. We checked models for the assumption of proportionality of hazards using the *cox.zph* function.

## Results

### SBPV infection course

**Fig. 1:**
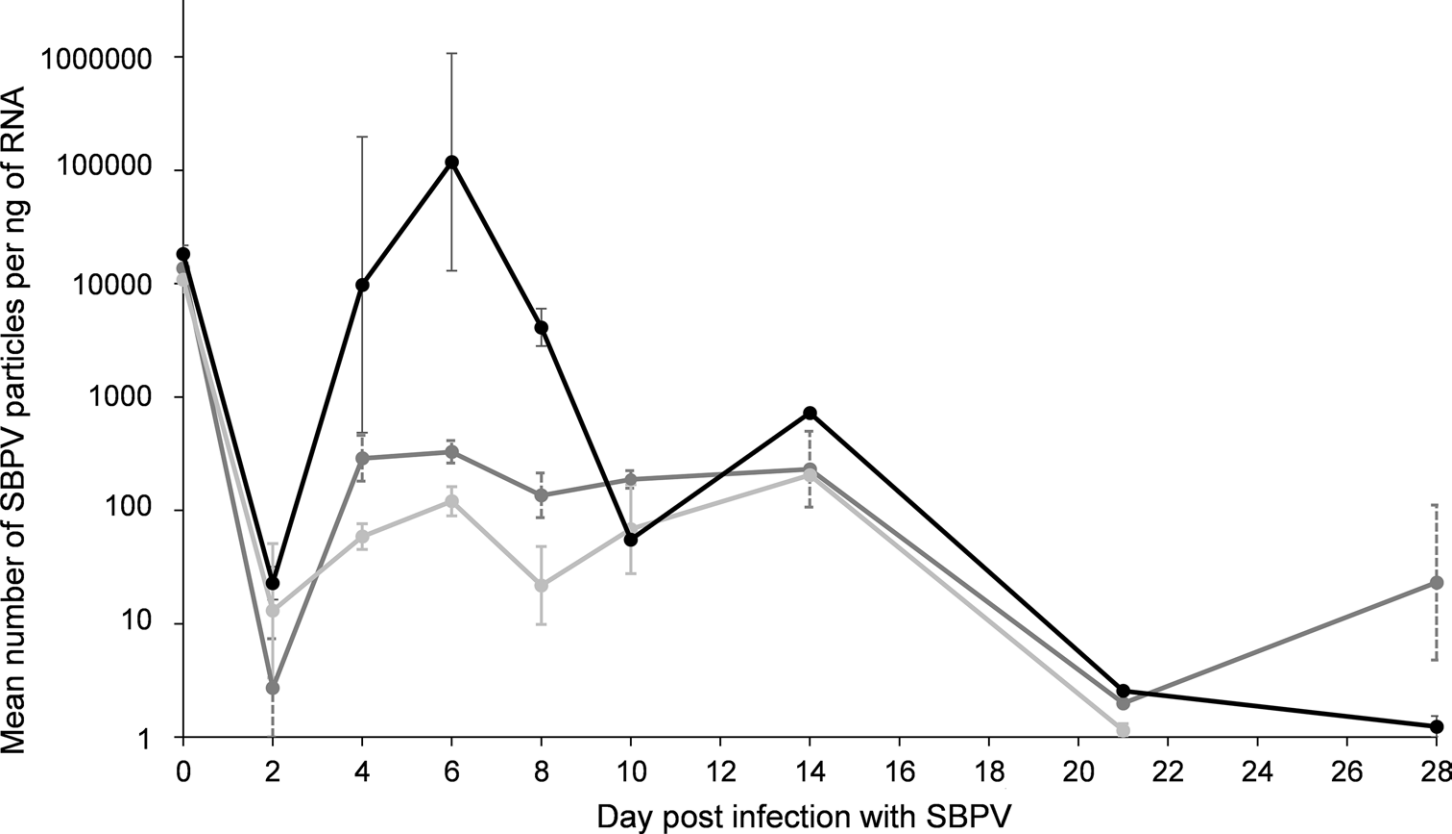
Mean slow bee paralysis virus (SBPV) load (viral copies per ng of RNA) per colony over a 28-day time course of SBPV infection in the guts of *B. terrestris*. Three guts per colony were analysed per time point on day 0, 2, 4, 6, 8, 10, 14, 21, and 28 (note; only 1 or 2 samples were analysed per colony at days 14 and 21, and no bees remained on day 28 to kill for colony C). Standard error bars are included (note; not for days 14 and 21 when there were less than two data points. Key: colony A (*black*), colony B (*dark grey*) and colony C (*light grey*).* Asterisk* above a time point indicates a significant increase in viral load compared to day two, across all colonies (one-sided Kolmogorov–Smirnov tests). Note log scale used on Y axis

To confirm that SBPV replicates in bumblebees, we tracked SBPV in inoculated bees for 28 days (Fig. 1). Day post-inoculation is a significant factor determining log viral load (GLMM: *t* = −3.8_59.4_, *p* < 0.001). Bees killed on day zero, 2 h post-inoculation, had a mean viral load of 1.5 × 10^4^ copies per ng of RNA (ranging from 8 × 10^3^ to 2.5 × 10^4^ copies, *N* = 9), corresponding to a mean of 3 × 10^7^ copies per bee gut. There was a reduction in mean viral load between day zero and day two to less than 50 copies per ng of RNA (ranging from viral absence to 103 copies, *N* = 8). Compared to day two, viral loads were significantly higher on day four [mean = 3.4 × 10^5^ viral copies (*N* = 9), *D* = 0.9, *p* < 0.01], day six [mean = 10 × 10^5^ viral copies (*N* = 9), *D* = 0.9, *p* < 0.01], day eight [mean = 1.6 × 10^3^ viral copies (*N* = 9), *D* = 0.7, *p* = 0.01], day ten [mean = 1.4 × 10^2^ viral copies (*N* = 7), *D* = 0.7, *p* = 0.02] and day fourteen [mean = 3.8 × 10^2^ viral copies (*N* = 4), *D* = 1, *p* = 0.006] (Fig. 1), implying that the virus results in a replicating infection. Two bees out of 64, both from colony A, replicated the virus beyond the initial viral load, reaching viral loads of 3 × 10^6^ and 9.7 × 10^6^ per ng of RNA, on day four and six, respectively).

### Survival assays

**Fig. 2:**
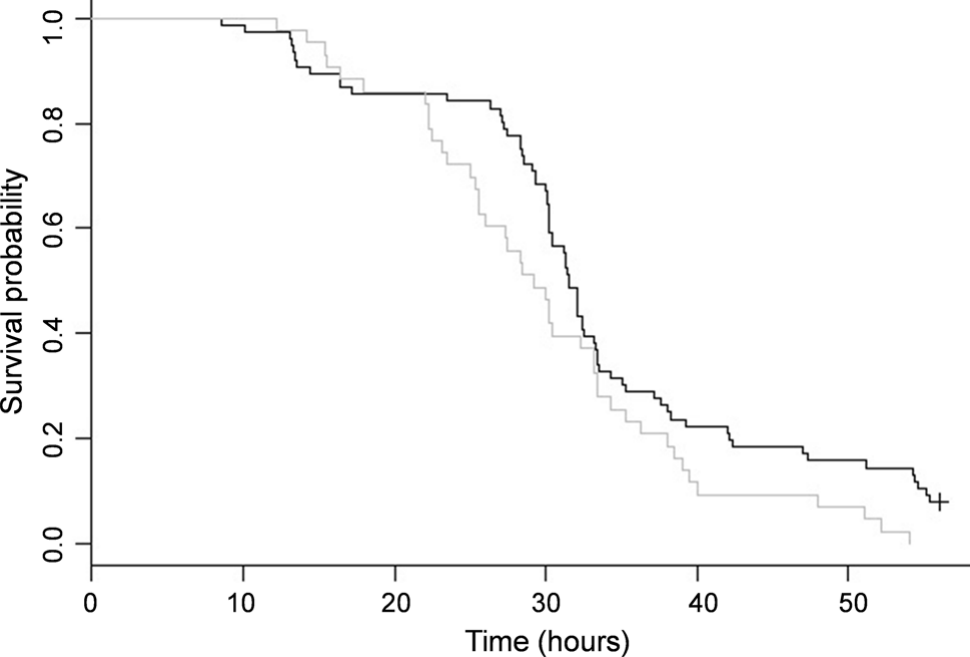
Kaplan–Meier survival curves for bees infected with slow bee paralysis virus (SBPV) (*grey*) and disease-free bees (*black*) during the starvation assay (contaminated controls excluded)

**Fig. 3:**
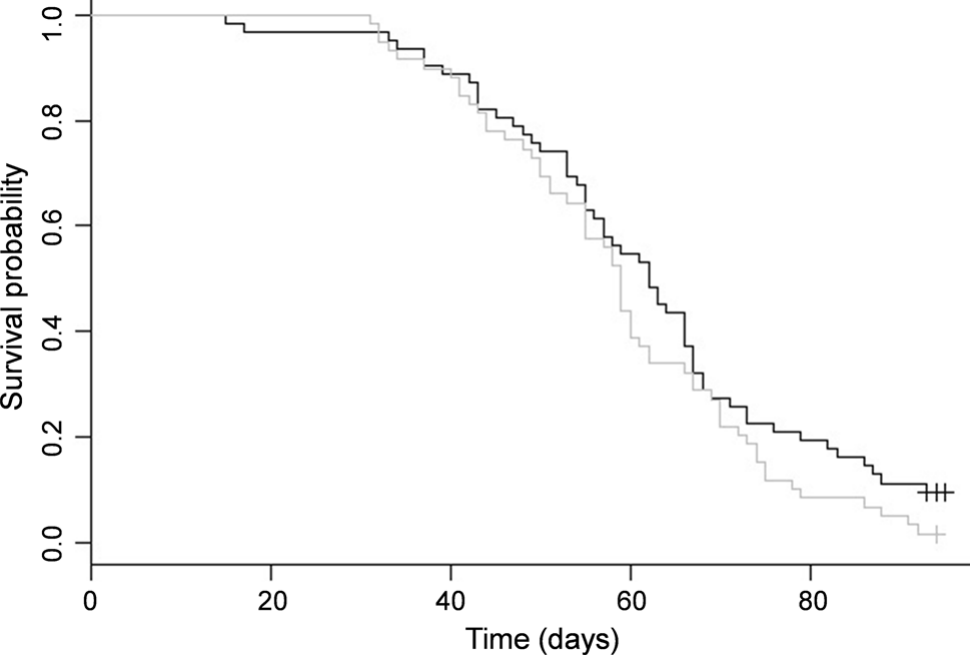
Kaplan–Meier survival curves for bees infected with slow bee paralysis virus (SBPV) (*grey*) and disease-free bees (*black*) during the satiated assay (contaminated controls excluded)

There is a significant difference in survival between SBPV-positive and SBPV-negative bees when stressed by starvation (Fig. 2; *N* = 119, log-rank test: *χ* ^2^ = 4.5_1_, *p* = 0.03) with a median difference in survival of 2.3 h, but no difference under satiated conditions (Fig. 3; *N* = 121, log-rank test: *χ* ^2^ = 2.3_1_, *p* = 0.1). Longevity did not vary according to host colony in our experiment (log-rank test for starvation: *χ* ^2^ = 4.2_2_, *p* = 0.1, and satiated conditions: *χ* ^2^ = 2.2_2_, *p* = 0.3).

#### SBPV infection status

SBPV inoculated bees are able to clear infection: with 34 of 77 starvation bees clearing infection before being killed at day 10 post-inoculation, and 14 of 73 satiated bees clearing infection before they died naturally at various ages. Interestingly, there is a significant difference in virus clearance rates between the two assays (test of proportions; *χ* ^2^ = 9.6_1_, *p* < 0.01). SBPV appears to be highly contagious by a transmission route other than direct inoculation. Even though the experimental set-up prevented any direct contact between control bees and SBPV, 26 of 68 starvation control bees and 21 of 69 satiated control bees became contaminated with SBPV during the experiment. Note, there is no significant difference in number of contaminated controls between assays (test of proportions; *χ* ^2^ = 0.4_1_, *p* = 0.5). Thus, for the analysis we compared infected versus uninfected bees, rather than inoculated versus control bees (table S3). Based on qualitative electrophoresis gel data, it is notable that inoculated bees have a range of viral loads, while contaminated control bees generally have a low viral load (figure S3).

It is important to note that the route of infection—whether inoculation or contamination—does not affect the overall results. Bees positive for SBPV had reduced longevity under starvation conditions when all bees are included in the analysis [median difference in survival = 1.34 h; *N* = 145, HR = 1.4 (1.0–2.0), *χ* ^2^ = 3.8_1_, *p* = 0.05], if all control bees are removed from analysis [median difference in survival = 3 h; Cox regression: *N* = 77, HR = 1.87 (1.1–3.16), *χ* ^2^ = 5.42_1_, *p* = 0.02], or if only infected controls are removed (median difference in survival = 2.3 h (Table 1) (table S3). Although interesting, we do not know enough about the route of infection or the dose of SBPV received by contaminated controls to draw any conclusions, and have thus excluded them from the main survival analysis for both starvation and satiated conditions.

**Table 1:** Cox regression models comparing survival of slow bee paralysis virus (SBPV)-infected bees to SBPV-free bees under starvation conditions and favourable conditions. Colony was fitted as a random effect

Variable	Regression coefficient (b)	SE (b)	*p* value	HR (e^b^)	95% CIs for HR	
Starvation conditions (*N* = 119, events = 113)					Lower	Upper
Infection status (0 = not infected, 1 = infected)	0.4	0.2	0.03**	1.6	1.0	2.4
Colony A				0.8	0.2	2.6
Colony B	n/a	n/a	0.12	0.9	0.3	3.0
Colony C				1.3	0.4	3.0
Fat ratio	−0.8	0.5	0.07*	0.4	0.2	1.0
Body size (wing)	0.3	0.4	0.4	1.4	0.6	3.4
Overall model	Likelihood ratio = 13.9_3.94_			*p* = 0.007**		
Favourable conditions(*N* = 121, events = 114)						
Body size (wing)	−1.0	0.4	0.01**	0.4	0.2	0.8
Infection status (0 = not infected, 1 = infected)	0.2	0.2	0.2	1.3	0.9	1.9
Batch number (1,2,3,4)	0.1	0.1	0.2	1.2	1.0	1.4
Colony A	n/a	n/a	0.3	1.2	0.4	3.8
Colony B				1.0	0.3	3.2
Colony C				0.8	0.2	2.7
Overall model	Likelihood ratio = 10.3_3.94_			*p* = 0.03**		

*HR* hazard ratio, *CIs* 95% confidence intervals for HR. Variables underlined are included in the final model, variables that are not underlined were included in maximal models but removed by model simplification

** Indicates significance at 95% and *at 90%. Overall model fit refers to the minimum adequate model

#### Starvation conditions

Of the 119 workers in the starvation assay (excluding the infected controls), 113 subsequently died during the experiment. A maximal Cox proportional hazards regression model included the effects of infection status, body size, relative fat content and host colony, as well as an interaction between infection status and fat content, on the survival of bees. Infection status was significant (hazard ratio (HR) = 1.6, 95% CIs [1.0, 2.3], *χ* ^2^ = 4.5_1_, *p* = 0.03). Based on the hazard ratio, SBPV-infected bees are 1.6 times more likely to die at a given time point compared to uninfected bees. The data also suggests that the risk of death decreases with increasing relative fat content (HR = 0.4, 95% CIs [0.2, 1.0], *χ* ^2^ = 3.3_1_, *p* = 0.07). Host colony was fitted as a random effect, and although not significant, it was retained as an important variable within the model. There was no interaction between fat content and body size with infection status. All variables satisfied the assumption of proportional hazards (table S4a). The overall model was significant (likelihood ratio test: 13.9_3.94_, *p* = 0.007) (Table 1).

As a result of the ability of inoculated bees to clear infection, the data can be divided into three groups: (1) SBPV-positive bees that were inoculated and maintained infection (*N* = 43), (2) SBPV-negative bees that were inoculated but cleared infection (*N* = 34), and (3) SBPV-negative control bees (*N* = 42). Note, we assume 100% of inoculated bees initially became infected based on qPCR data from our infection time course (Fig. 1). Interestingly, bees that cleared infection did not differ in survival from control bees; but both these SBPV-negative groups differ in survival from SBPV-positive inoculated bees (Table 2 and figure S4: note, these also includes pairwise comparison with contaminated controls, indicating that survival of SBPV-positive contaminated control bees does not significantly differ from survival of SBPV-positive inoculated bees).

**Table 2:** Kaplan–Meier log-rank test results between the survival probabilities of four groups of bees under starvation conditions: inoculated (+) are SBPV inoculated bees that maintained infection, inoculated (−) are inoculated bees that cleared infection, controls (+) are control bees that became infected indirectly, and controls (−) are control bees that remained clean

	Inoculated (+)	Inoculated (−)	Controls (+)
Inoculated (+) (*n* = 43)			
Inoculated (−) (*n* = 34)	*χ* ^2^ = 4_1_,* p* = 0.045 **		
Controls (+) (*n* = 26)	*χ* ^2^ = 0.4_1_,* p *= 0.506	*χ* ^2^ = 1.3_1_, *p* = 0.2	
Controls (−) (*n* = 42)	*χ* ^2^ = 3.7_1_,* p* = 0.077 *	*χ* ^2^ = 0.1_1_, *p* = 0.7	*χ* ^2^ = 0.7_1_, *p* = 0.415

The number of bees in each group is recorded in parentheses

** Indicates significant difference in survival between two groups at 95% and * at 90%

#### Satiated conditions

Of the 121 workers in the satiated assay (excluding the infected controls), 114 subsequently died during the experiment. Infection had no significant effect on survival but was retained in the model as an important variable. Batch had no effect on survival and was removed from the model by model simplification. Only body size had a positive significant effect on survival (HR = 0.4 CIs [0.2, 0.8], *χ* ^2^ = 6.49_1_, *p* = 0.01) (Table 1). Host colony was not significant but was retained as an important variable within the model. The overall model was significant (likelihood ratio test: 10.3_3.94_, *p* < 0.03). All variables satisfied the assumption of proportional hazards (table S4b). Under satiated conditions there was no difference in survival between the four groups mentioned above [(1) SBPV-positive bees that were inoculated and maintained infection (*N* = 59), (2) SBPV-negative bees that were inoculated but cleared infection (*N* = 14), (3) SBPV-negative control bees (*N* = 48)] and (4) SBPV-positive contaminated control bees (*N* = 21) (*χ* ^2^ = 2.4_3_, *p* = 0.5).

### Colony effects

There are colony differences in susceptibility to SBPV, with colony A showing significantly higher titres compared to the other two colonies (Fig. 1, GLMM; *t* = 5.3_5.8_, *p* < 0.001). In addition, colony A was significantly less able to clear infection in the starvation assay (*χ* ^2^ = 12.1, *p* = 0.002): post-starvation (i.e. day 10 post-infection) 44% (*N* = 77) of inoculated bees were clear of infection—in colony A only 19% (5 of 26) of inoculated bees had cleared infection by day 10, while 60% (15 of 25) and 58% (15 of 26) of individuals had cleared the infection in colony B and C, respectively. In the longevity assay, few bees had cleared infection at the time of death [12.5% (*n* = 24), 27% (*n* = 30) and 15.8% (*n* = 19) in colony A, B and C, respectively, with no significant differences between colonies (χ^2^ = 1.9, *p* = 0.38).

## Discussion

Slow bee paralysis virus is widespread and prevalent across bumblebee species in the UK and occurs in apparently healthy foraging individuals (McMahon et al. 2015). Our results demonstrate that SBPV has the potential to exert a hidden cost on its host *B. terrestris*. Under satiated conditions, when *B. terrestris* have unlimited access to food, SBPV infection has no effect on longevity; while, under starvation conditions, the longevity of infected bees is significantly reduced—they are 1.6 times more likely to die at each time point. Under lab conditions, infected bees survived on average 2.3 h less under starvation. In the wild, periods of starvation are likely to occur frequently for bumblebees due to adverse weather conditions such as high winds, rain or cold weather. Unlike honeybees, bumblebees do not have significant honey stores in their nests. A decreased survival of a median of 2.3 h would thus be ecological significant for bumblebees in the wild (Brown et al. 2000); more importantly, this effect would most likely be exacerbated under real life conditions where individuals need to expend additional energy for, e.g. thermoregulation and flight, therefore the impact of viral infection may be underestimated here.

The experimental starvation conditions used in this experiment were designed to imitate natural situations where poor weather can prevent bees foraging for extended periods. Both pollen and nectar are critical energy resources for bumblebees, and colonies suffering from an energy shortfall are less effective at protecting the colony from predators, social parasites (Cartar and Dill 1991) and pathogens (Alaux et al. 2010; Moret and Schmid-Hempel 2000). As our results show, the virulence of a viral pathogen, which may appear asymptomatic under benign conditions, is also unmasked under such energy-limited conditions. Given such synergistic effects of environmental stress and disease there is clearly the need for more consideration of spillover effects of viral disease between managed bees and wild pollinators.

Condition-dependent virulence has been demonstrated in bumblebees for the gut parasite *Crithidia bombi,* which also decreases survival under starvation (Brown et al. 2000). *C. bombi* additionally exerts fitness costs during stressful times in the bumblebee life cycle, such as queen hibernation and colony foundation (Brown et al. 2003). For bee viruses, condition-dependent virulence of DWV has been demonstrated under pesticide stress (Di Prisco et al. 2013) as well as nutritional stress (Degrandi-Hoffman et al. 2010). In some sense, co-infection of viruses with the ectoparasitic honeybee mite *V. destructor* is probably the best documented example of condition-dependent virulence [SBPV (Carreck et al. 2010), DWV (Martin et al. 2012), ABPV (Genersch et al. 2010) and IAPV (Di Prisco et al. 2011)]. However, the mechanisms of increased virulence in this case are more complicated. Although *V. destructor* weakens the bees and may cause immunosuppression (Nazzi et al. 2012; Yang and Cox-Foster 2005) similar to other environmental stresses, the mite itself increases viral virulence in its capacity as a virus vector (Martin 2001).

It is probable that, like *C. bombi*, other condition-dependent pathogens of bumblebees such as SBPV will affect colony fitness beyond mere survival to starvation. Clearly these individual and colony effects can lead to population level responses and pathogens are well documented to exert large effects on their host’s ecology (e.g. Anderson and May 1981; Hatcher et al. 2006). Theoretical models and eventually species specific simulation models would be useful in determining the likely population level effects of the individual impact of condition-dependent virulence under variable resource conditions.

The precise mechanism by which SBPV reduces host longevity under starvation conditions is unclear. It is possible that infection induces a costly response by the host immune system; in this scenario, the host is unable to maintain a defence against the virus when resources are withheld, resulting in increased virus virulence and reduced lifespan (Moret and Schmid-Hempel 2000). It is also possible that infection reduces the resources in the gut that are available to the bee, or inhibits uptake of resources, as may be the case for trypanosomal gut parasites (Gorbunov 1987, 1996; Jensen et al. 1990). Such effects may be exacerbated by any damage caused by viral replication within its host, with the pathogen’s virulence likely to be determined by a combination of these factors.

Bees with higher body fat have reduced risk of death under starvation conditions, while body size itself has no effect. The fat body is key for immunity and longevity; it is the main site of energy and protein storage, synthesis of immunoproteins, and vitellogenin synthesis involved in longevity (Amdam and Omholt 2002). In *B. terrestris* workers, fat body increases with age (Moret and Schmid-Hempel 2009). As the bees in this assay were of mixed age (within 3 weeks of each other), although randomised across treatments, it is possible the relationship between fat body and survival could be linked to age. Body size had no effect on longevity over short-term starvation but had a significant positive effect on longevity under satiated conditions. Body size is determined by conditions during larval development. Maintaining brood at 30 °C requires high energy consumption (Heinrich 1974). When resources are low, workers cease incubating and the brood temperature drop, which slows the development and potentially causes developmental defects (Barrow and Pickard 1985). Low body size and body fat are both symptoms of a lack of resources and reduce individual longevity, which could have consequences at a colony and population level.

Many measured immune defences decrease with age in *B. terrestris* workers, i.e. antibacterial activity, encapsulation and melanisation, haemocyte concentration and phenoloxidase activity, with declines seen within a biologically relevant age range (Doums et al. 2002; Moret and Schmid-Hempel 2009). It is interesting that the ability of bees in the starvation assay to clear viral infection is significantly higher than for bees in the satiated assay. Because of the mixed age of starvation bees it is important to consider how immunosenescence could influence viral clearance rates. The starvation bees were older (on average) at the time point of inoculation because of the age range 0–3 weeks (1–21 days old), while satiated bees were all inoculated at 6 days old. Thus, a higher viral clearance rate in older bees is contrary to the immunosenescence reported for the aspects of humoral and cellular immune defences in *B. terrestris,* mentioned above. However, not all immune measures decrease with age, e.g. fat body (Moret and Schmid-Hempel 2009). It is conceivable that anti-viral defences against oral-faecal infections might be stronger in older bees. In addition, in contrast to individual age, some immune measures increase with colony age. Moret and Schmid-Hempel (2009) found that *B. terrestris* workers born when the colony is young have lower concentration of haemocytes and lower PO activity than those born later in the colony life cycle. As starvation bees emerged at a later point in each colony’s life cycle, compared to satiated bees, increased immunity with colony age could also explain why starvation bees cleared infection at significantly higher rates. It is clear that experiments dedicated to studying immunosenescence of anti-viral defences in social insects are needed to understand the impact of viral infections on wild populations.

The dose of SBPV used in this study came from a natural infection and is high enough to cause an initial infection in the majority of inoculated bees, with condition-dependent effects on longevity. The infection persisted up to 95 days in our bees kept in satiated conditions, which would mean a life-long infection for worker bees in the wild. However, our data show a significant variation between colonies in viral replication levels (across several orders of magnitude at day four and six post-infection) and their ability to clear infection, suggesting a genetic basis for defence that is likely to be reflected in wild bumblebees.

Over 20% of the control bees became infected with SBPV indirectly. While contamination via plastic-ware cannot be categorically ruled out, this raises the possibility that SBPV may be an airborne pathogen. In addition, Graystock et al. (2016) recently showed that commercial irradiated pollen can still contain pathogens, and although they did not test for SBPV this is a possibility. It is noteworthy that there was no difference in survival of SBPV-infected bees, whether they were orally inoculated with a 5 µl dose or indirectly infected; it appears that SBPV is highly transmittable at low doses.

In summary, we have demonstrated that a common honeybee and bumblebee pathogen, that may appear asymptomatic in field collections and under optimal lab conditions, exerts a fitness cost on bumblebees under adverse conditions. Our results show the importance of examining subtle fitness effects when assessing a pathogen’s effect on its host. Additionally, we found that larger bumblebees—indicating energy-rich conditions during larval growth—had a higher longevity and individuals that survived starvation conditions for longer had larger fat reserves. Providing good forage opportunities for pollinators may thus directly contribute to their longevity and resistance to stressful conditions. This highlights the importance of providing forage opportunities for pollinators throughout the season, as laid out, for example, in the UK’s National Pollinator Strategy (DEFRA 2014). A wider uptake of conservation measures in land management under schemes such as the Countryside Stewardship in the UK could directly impact longevity and disease tolerance in pollinators.

Conditions in the lab are extremely favourable, e.g. individual lifespan under laboratory conditions exceeds the natural lifespan of worker bees in the field (Schmid-Hempel and Heeb 1991), such that laboratory studies can underestimate the impact of a pathogen. The use of sentinel species in studies of a multi-host-virus system may misrepresent the true impact of a pathogen on wild pollinator populations, as hosts can vary in their susceptibility to viruses (McMahon et al. 2015). In addition, estimates of virus prevalence are believed to be underestimated (McMahon et al. 2015). Thus, the impact of SBPV on natural populations may be greater than predicted. Indeed, it is possible that SBPV does affect longevity under satiated nutritional conditions in the wild, as workers would face additional ecological and environmental stressors such as inclement weather, energetically costly foraging and exposure to pesticides and pollutants. More broadly, this demonstrates that impact assessments of emerging multi-host pathogens, such as West Nile Virus in the USA (Kilpatrick 2011) need to take into account the pathogen’s ecology rather than narrowly focusing on the most tractable laboratory model system.

## Electronic supplementary material

Below is the link to the electronic supplementary material.

Supplementary material 1 (DOCX 338 kb)
