# Supplementary material for: Condition-dependent virulence of slow bee paralysis virus in Bombus terrestris: are the impacts of honeybee viruses in wild pollinators underestimated?
Source: Oecologia. 2017 Mar 30;184(2):305–15. doi: 10.1007/s00442-017-3851-2 (PMC5487845; doi:10.1007/s00442-017-3851-2)
Supplement: Supplementary file 1 — Supplementary material 1 (DOCX 338 kb) [file 442_2017_3851_MOESM1_ESM.docx]

**Manley, Boots and Wilfert (2016) Condition-dependent virulence of Slow Bee Paralysis Virus in *Bombus terrestris*: Are the impacts of honeybee viruses in wild pollinators underestimated?**

**Supplementary material**

Table S1. The molecular and thermal conditions used for each pair of primers,

| Primers | Assay mix | | | | | | | | Thermal cycling | | | Amplicon size (bp) |
| --- | --- | --- | --- | --- | --- | --- | --- | --- | --- | --- | --- | --- |
|  | 5x buffer (ul) | MgCl_2_ (mM) | dNTPS (mM) | Primer F(uM) | Primer R (uM) | Taq (ul) | Template (ul) | Final volume (ul) | Denaturing (Temp\|Min) | Replication  (Temp\|sec) | Elongation (Temp\|min) |  |
| SBPV | 4 | 2.5 | 0.2 | 0.4 | 0.4 | 0.15 | 2 | 20 | 94\|5 | ^X 35^  94\|15  55\|30  72\|20 | 72\|7 | 915 |
| ABPV | 4 | 2.5 | 0.2 | 0.4 | 0.4 | 0.15 | 2 | 20 | 94\|5 | ^X 35^  94\|15  55\|30  72\|70 | 72\|7 | 1034 |
| DWV | 4 | 2.5 | 0.2 | 0.4 | 0.4 | 0.15 | 2 | 20 | 94\|5 | ^Touchdown PCR^  ^X10^  94\|15  62\|30  72\|60  ^X30^  94\|15  [Fst-62\|30  Lst-52\|30]  72\|60 | 72\|7 | 644 |
| BQCV | 4 | 2.5 | 0.2 | 0.4 | 0.4 | 0.15 | 2 | 20 | 95\|2 | ^x35^  95\|15  60\|30  72\|60 | 72\|7 | 986 |
| AK (Arginine Kinase) | 3 | 2.5 | 0.2 | 1 | 1 | 0.15 | 3 | 15 | 94\|5 | ^x35^  94\|15  55\|30  72\|20 | 72\|7 | 263 |

Table S2: Primer Sequences

| Primer name | Sequence 5’-3’ |
| --- | --- |
| SBPV 774F  SBPV 1689R | GAGATGGATMGRCCTGAAGG  CATGAGCCCAKGARTGTGAA |
| ABPV 5088F  ABPV 6122R | CyATGGACACACCCTATGTG  CGCCATTTTGGTACTTCTCC |
| DWV 8577F *  DWV 7933R | AACTGGCGAYCATACTCAGC  WCCAGGCACMCCACATACAG |
| BQCV 4119F  BQCV 5376R | TCCyCCAGTTCAACCATCTA  AACGTTGCCTAGrTTCGTCA |
| ACTB F  ACTB R | TGACAAGCATCCACCAAAAG  TCGTCGATCAGTTTCTGCTG |

*DWV primers not-specific to any strain of DWV, taken from [Wilfert *et al.* (2016)](#_ENREF_1)

Table S3**.** The number of bees in each experimental group, followed by the groups that were used in the survival analysis. Note: we tested infected vs uninfected bees, rather than inoculated vs control bees because of the number of bees that cleared or gained infection within each group. Note 2: 68 of the original 73 control bees in the starvation assay were used in the experiment because five bees died prior to the starvation assay (day 10). Importantly, the main results remain unchanged whether contaminated/cleared individuals are removed or not. The result in bold at the end of the table is the main result we report in the paper.

|  | Starvation | | Survival analysis (Cox proportional hazards regression for infection variable reported) | Satiated | | Survival  analysis |
| --- | --- | --- | --- | --- | --- | --- |
|  | **Inoculated** | **Controls** |  | **Inoculated** | **Controls** |  |
| Total bees | 77 | 73 |  | 73 | 69 |  |
| Used in experiment | 77 | 68 | n/a | 73 | 69 | n/a |
| Bees Cleared infection | 34 | n/a | n/a | 14 | n/a | n/a |
| Bees gained infection | n/a | 26 | n/a | n/a | 21 | n/a |
| Total bees without positive controls | 77 | 42 | n/a | 73 | 48 | n/a |
|  | **Infected** | **Uninfected** |  | **Infected** | **Uninfected** |  |
| Total bees | 69 | 76 | Sig median difference in survival = 1.34hr; N = 145, HR = 1.4 (1.0 – 2.0), χ^2^ = 3.8_1_, p = 0.05 | 80 | 62 | Not sig |
| Total bees without controls | 43 | 34 | Sig median difference in survival = 3hr; Cox regression: N = 77, HR = 1.87 (1.1-3.16), χ^2^ = 5.42_1_, *p* = 0.02) | 59 | 14 | Not sig |
| **Total without positive controls** | **43** | **76** | **Sig median difference in survival = 2.3hr: Cox regression: N = 119, HR = 1.6,** (1.0 – 2.3), χ^2^ = 4.5_1_, p = 0.03 | **59** | **62** | **Not sig** |

Table S4a and b: The output from testing the assumption of proportional hazards for the Cox proportional hazards model (using coxph function) under starvation (a) conditions and satiated (b) conditions. Rho is the Pearson product moment correlation between the scaled Schoenfeld residuals and log(time) for each variable. The final row contains the global test for all the interactions tested at once. A p value <0.05 shows a violation of the proportionality assumption.

**a.**

| **Variable** | **rho** | **Chi sq** | **p** |
| --- | --- | --- | --- |
| Infected | -0.026 | 0.079 | 0.778 |
| Fat ratio | -0.009 | 0.006 | 0.940 |
| Colony A | 0.221 | 0.359 | 0.549 |
| Colony B | 0.117 | 0.087 | 0.768 |
| Colony C | -0.310 | 0.352 | 0.553 |
| Global | n/a | 10.421 | 0.064 |

**b.**

| **Variable** | **rho** | **chisq** | **p** |
| --- | --- | --- | --- |
| Infected | 0.072 | 0.611 | 0.435 |
| Wing | -0.067 | 0.569 | 0.450 |
| Colony A | -0.065 | 0.020 | 0.888 |
| Colony B | 0.110 | 0.068 | 0.794 |
| Colony C | -0.043 | 0.017 | 0.896 |
| Global | n/a | 2.156 | 0.827 |


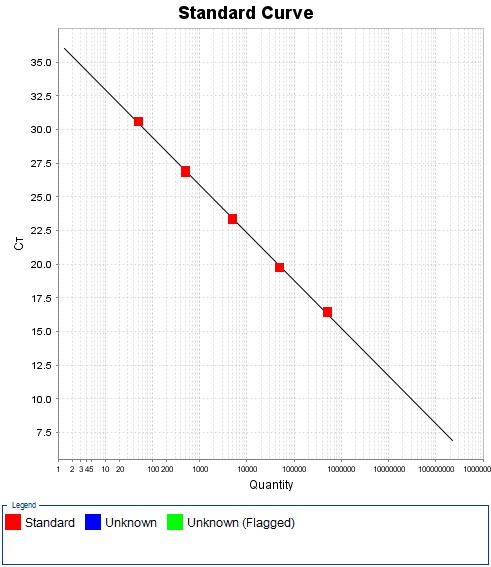


Figure S1. Standard curve for SBPV; Slope = -3.5, Y-inter: 36.5, R2 = 1, eff% = 91.8. Diagram from StepOne Software v 2.3

Figure S2. Cq values of individual bees in the gut time course for both Slow bee paralysis virus (SBPV) (black) and Actin Beta (ACTB) (grey) confirming extraction of a valid biological template and demonstrating that ACTB Cq stayed stable relative to SBPV Cq.

Figure S3. Electrophoresis gel picture of an SBPV PCR product (915 bp fragment) on a number of inoculated and control bees from the starvation assay. Unlabelled wells are inoculated bees; the bands with an asterisk are inoculated bees positive for SBPV. A1-A5, B1-B5 and C1-C5 wells are control bees from colonies A, B and C, respectively; + identifies controls positive for SBPV.


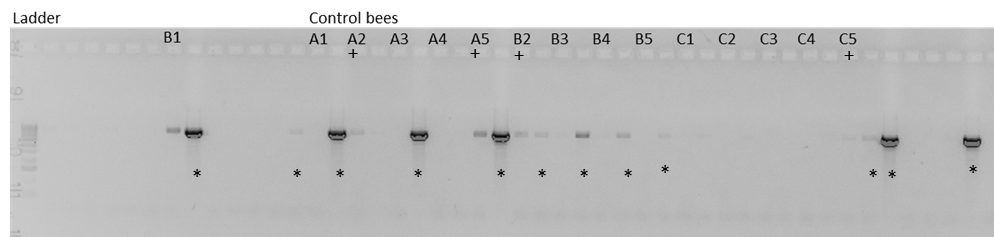


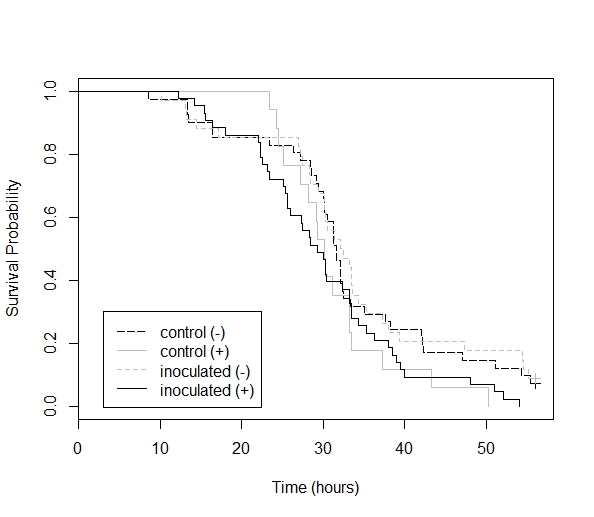


Figure S4. A comparison of the survival probability curves between four groups of bees in the starvation assay: Inoculated (+) are bees that were still infected at day 10 post inoculation (p.i.), Inoculated (-) are bees that were inoculated but cleared infection by day 10 p.i., Controls (-) are clean control bees and control (+) are infected controls.

References

Wilfert, L., Long, G., Leggett, H.C., Schmid-Hempel, P., Butlin, R., Martin, S.J.M. & Boots, M. (2016) Deformed wing virus is a recent global epidemic in honeybees driven by *Varroa* mites. 351, 6273:594-597 doi:
